# Can the kidney volume help to differentiate the types of rejection before biopsy?

**DOI:** 10.22088/cjim.10.1.11

**Published:** 2019

**Authors:** Mohammad Yazdani, Nasser Ghaemian, Soraya Khafri, Farshid Oliaei

**Affiliations:** 1 Student Research Committee, Babol University of Medical Science, Babol, Iran; 2 Cancer Research Center, Health Research Institute, Babol University of Medical Sciences, Babol, Iran; 3 Infertility and Health Reproductive Research Center, Health Research Institute, Babol University of Medical Sciences, Babol, Iran; 4 Cellular and Molecular Biology Research Center, Health Research Institute, Babol University of Medical Sciences, Babol, Iran

**Keywords:** Kidney transplantation, Kidney volume, AMR, CMR, Ultrasonography

## Abstract

**Background:**

The aim of this study was to use the volume of the graft as an adjunct tool for better decision making.

**Methods:**

Kidney transplanted patients with acute azotemia and documented volume and finally a biopsy were enrolled in this study‌. Graft volumes between rejected patients (antibody-mediated rejection {AMR} and cell - mediated rejection {CMR}) and ‌non rejected but azotemic patients were compared.

**Results:**

A total of 76 patients were enrolled in this study ‌(45 case and 31 control‌). 53.3% of the case group were‌ (AMR)‌ and 46.7% belonged to ‌(CMR). There was no difference between kidney volume according to age or sex. But the case group had a significantly bigger volume than controls (253.09 cm^3^ and 186.45 cm^3^; p< 0.001). In addition, there was a significant difference between the volumes of AMR kidneys with CMR and controls ‌(286.24+66.70‌, 224.08+76.79 and 186.95+39.92; P=0.003 and p<0.001, respectively), but not between CMR and controls ‌(P=0.067). A cutoff point of 200 cm^3^ was determined as rejection with sensitivity and specificity of 70% and a cutoff point of 250 cm^3^ could be used as AMR cut off with sensitivity of 76% and specificity of 70%.

**Conclusion:**

There was a significant difference in volume between rejection and control group and between AMR and CMR. So, kidney volume determination is an easy and valuable tool to help the clinician to have a more rapid and better decision making.

Kidney transplantation (KT) is the optimal treatment of end stage renal disease (ESRD) (1). Acute kidney injury (AKI) in KT is common and has a variety of causes like rejection, drug toxicity, tubule necrosis, infection and obstruction and biopsy has a great role to differentiate them from each other and rejection is the most important cause of graft loss (2, 3). In the simplest way, AKI is presented with creatinine rising but kidney biopsy is still the gold standard in diagnosing the exact cause of AKI in grafts (4), renal sonography and biopsy were excluded from the study. According to the last classification of Banff pathologic criteria (2007) rejections were divided into 3 groups: 1- cell mediated rejection (CMR), 2- antibody mediated rejection (AMR), and 3- AMR/CMR. However, the transplant biopsy is limited by the risk of renal injury and a fundamental dependence on descriptive consensus classification, so precise individual interpretation is not fully met by the contemporary transplant pathology. Thus, a great effort to find different imaging in place of biopsy is going on (5-7). Ultrasonography (US) either as a gray scale or color Doppler has an expanded use in AKI to detect enlargement, collections, perfusion and venous congestion.

Application of kidney volume by US, was not so common till now and a few studies had noted the increase in volume during rejection (8, 9). In the past decade, with the emergence of antibody mediated rejection (AMR) and its difference with cell mediated rejection (CMR) in diagnosis and treatment, a complementary test feels to be needed to clear the vague results of pathology. 

 The aim of this study was to use the 3D US in two types of rejections and in control group to detect the differences between them. It may help the pathology in unclear cases.

## Methods

**Patients’ Data: **Patients who referred to Shahid Beheshti Hospital from 2007 to 2014 with at least 6 months post-transplantation period and acute renal dysfunction (acute increase in creatinine of more than 50% ), were included in this study. According to the biopsy results, the azotemic patients were divided into rejected (case) and non-rejected (control) groups and also we had two rejected groups: namely the, AMR and CMR. All the grafts were received from living donors and the immunosuppressive protocol was composed of cyclosporine, mycophenolate and prednisolone without any recent changes before admission. Patients did not receive steroids before US. Control group consists of azotemic patients with any other diagnoses like drug toxicity or non-specific inflammation but no rejejction or infection. Informed consent was taken from patients for biopsy and the study was approved by the Ethics Committee of Babol University of Medical Sciences. Patients with positive urine culture indicative of acute pyelonephritis and those with more than 3 days time span 

**Ultrasonography: **All the patients underwent US before Bx. The US was performed by one specialist who was not aware of the study using IU22 Philips (2012) probe convex 2-5 MHZ device and GE 500 (2002) Pro/probe 4 MHZ device . Patients were laid in supine position. Kidney volume was determined in cubic milliliter by non-homogeneous structures volume method (length * height * width * 0.523). Hydronephrotic cases were excluded from the study.

**Kidney Bx: **Biopsies were performed with an 18-gauge biopsy needle (Bard Peripheral Technologies, Covington, GA, USA) under the guide of US by automated method. Two core needle biopsies were taken from anterior parenchyma and the specimens were sent for light and immunofluorescence microscopy to one pathology center in Tehran. The results have been reported by at least two pathologists (not aware of the study).

**Statistical analysis: **SPSS Version 22 was used for statistical analysis. Kolmogorov-Smirnov test was used to evaluate the normal distribution of quantitative data. Quantitative variables were expressed as mean + SD. One–way ANOVA was used to evaluate the mean of kidney volume in different causes of acute rejection of renal transplant and control group. Independent t-test was used to assess the differences in kidney volume in male and female patients. Pearson correlation coefficient was used to assess the correlation of kidney volume and age. ROC- curve was used to determine the cutoff point of kidney volume. A p-value less than 0.05 was considered significant.

## Results

**Figure1:**
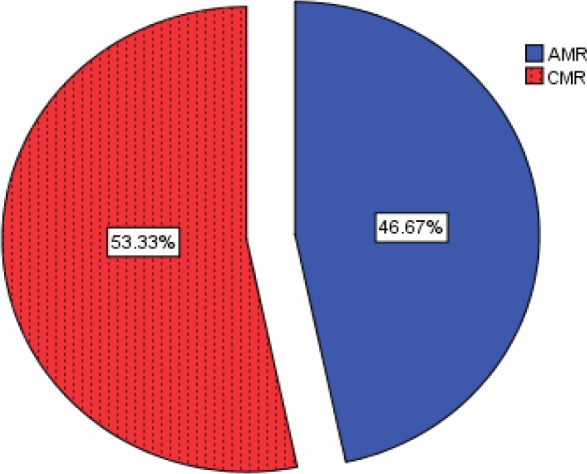
Frequency of acute rejection causes

A total of 76 patients participated in this study (45 cases and 31 controls). Different causes of acute rejection are represented in figure1. 

As explained before, the case group was divided further to AMR and CMR subgroups. Demographic parameters (age & gender) of three groups are represented in table1.

**Table1:** Comparison of demographic variables in three groups

**Groups Variables**	**Patients**		**Control**	**Pvalue**
	**AMR**	**CMR**		
**Gender**				
Male Female	13(25.5) 08(32.0)	16(31.4) 08(32.0)	22(43) 09(36)	0.791
Age(years)	43.1±12.55	38.71±10.05	41.03±12.95	0.474
**Diabetic**				
Yes No	04(36.4) 17(26.2)	3.0(27.3) 21(32.3)	04(36.4) 27(41.5)	0.782

The values are Mean±SD and n (%).

There was no relationship between kidney volume and demographic variables (Table 2).

**Table 2:** Relationship of demographic factors and kidney volume

**Variable**	**Gender**		**Age**
	**Male**	**Female**	
Volume	224.65±77.02	228.48±64.62	0.102
P-value	0.830		0.379

The values are Mean±SD and Pearson correlation coefficient.

**Figure 2:**
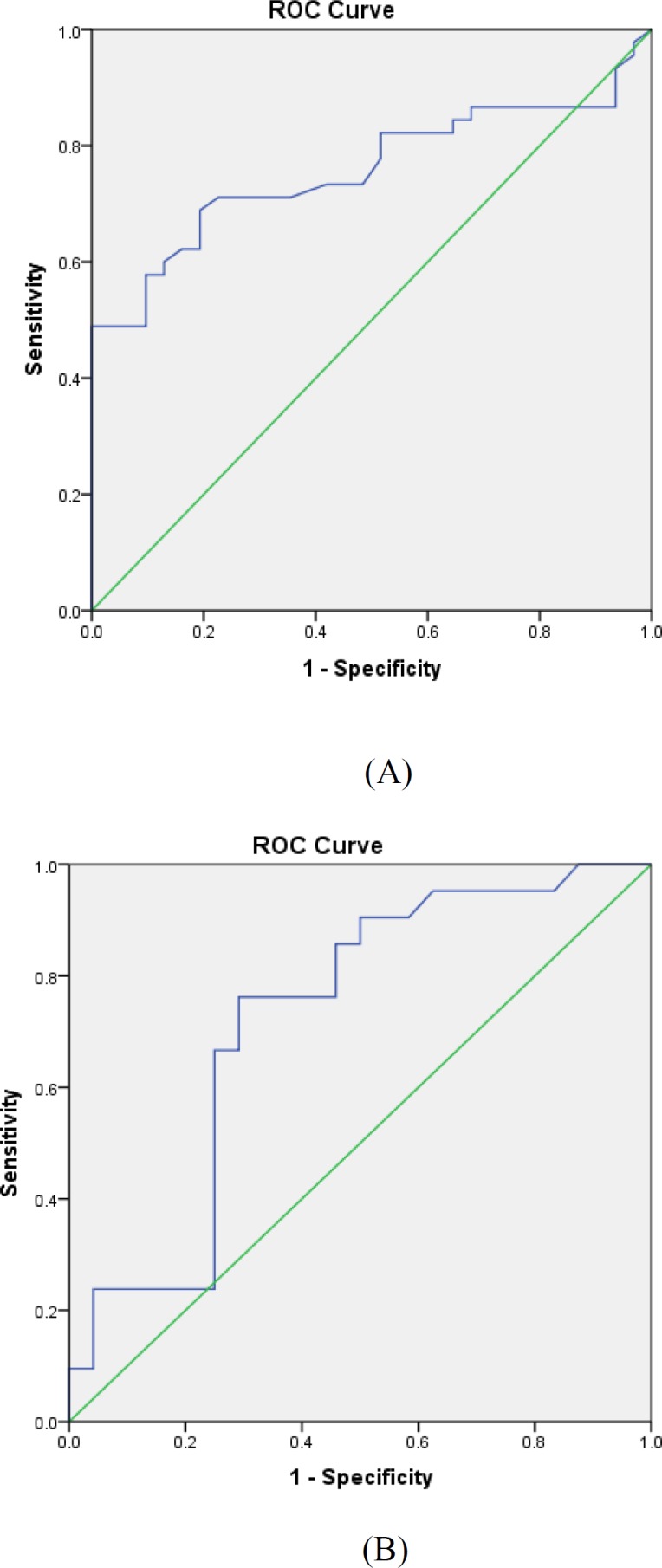
Accuracy of volume kidney in three groups. A: rejected cutoff point with sensitivity and specificity of 70% and accuracy of 0.76, B: the cutoff of volume in AMR and CMR

The volume of kidney in case group was significantly higher than in control group (253.09 and 186.45; respectively; p<0.001). The mean volumes of the AMR, CMR and control groups were 286.24+70, 224.08+76.74 and 186.95+39.92, respectively. There were significant differences between volumes of AMR with CMR and controls (P=0.003 and p<0.001, respectively); but not a significant difference is seen between CMR and control groups (pv=0.067). ROC curve was used to determine the accuracy and the cutoff point of kidney volume in three groups. The value of 200 cm^3^ was determined as rejected cutoff point with sensitivity and specificity of 70% and accuracy of 0.76 (fig. 2a). Figure 2b is the ROC curve about the cutoff of volume in AMR and CMR. It shows that the volume of 250 cm^3^ can be used as AMR cutoff point with sensitivity of 76% and specificity of 70%. Its accuracy was 0.72.

## Discussion

To our knowledge, this is the first study to compare the kidney volumes with different causes of acute rejection and normal variant. According to this study there was a statistically significant difference in volume between rejection and control group and on the other hand a great difference between AMR and CMR.

We found that AMR makes much bigger kidneys than CMR and this finding is justifiable to pathophysiologic events in acute antibody mediated rejection, because this type of rejection mostly involves small glomerular and pre-tubular vessels and leads to thrombosis, infarcts and necrosis; that is accompanied by infiltration of neutrophils and release of cytokines that make edematous inflammation. On the other hand, pathophysiological findings in acute cellular rejection show lymphocytic T-cell infiltration (mostly CD8+) and usually lead to reversible cytotoxic tubulitic; that is accompanied by infiltration of monocytes and macrophages and focal peritubular edema with few necrotic changes (10).

Hypertrophy after renal transplantation is considered normal (11). At the end of the second week after transplantation, an increase of kidney volume between 7-21% with mean of 16% is considered normal. The mean increase of kidney volume in successful transplantation was reported to be 22% at the end of third week (12). Any sudden changes in kidney volume in a short time can indicate acute rejection (12). The 10% increase in the cross sectional dimensions of the kidneys in the short time and the 20% increase of kidney volume within 5 days along with increase in serum creatinine level are symptoms of acute rejection (13). In a study conducted by Absy, they concluded that kidney volume would be constant during six months after transplantation and the kidney volume was significantly associated with renal function. They also observed renal hypertrophy in patients with diabetes and an increase of kidney volume in acute rejection (14). 

Furthermore, in Hricak’s study, an increase of kidney volume in acute rejection was detected by US and application of ultrasound was suggested in acute renal failure after transplantation (12). These results that seem to be ignored for about 3 decades are consistent with our results. In another study conducted by Frick et al., they found that an increase in kidney volume and decrease in echogenicity of renal pyramid in ultrasound findings is significantly consistent with biopsy results in patients who are under treatment for acute rejection (15). 

Application of volume rather than two dimensional section is because of our desire to have one number to make a cutoff point for rejection. In addition, it seems that by volume the false negative results of two dimensional detection can be avoided (self-experience). Krejci et al, in 2009 showed that edema in subclinical and borderline rejections (protocol biopsies) could be differentiated from normal by conventional US. But again, there was nothing about kidney volume and CMR or AMR (16). Pathology as the gold standard tool for AKI in transplantation, has misleading overlaps in some cases and it is due to a mixture of two types of rejection in some samples but it is important to determine the relative distribution of the two types (6). So, a volume of 200 cm² can be suggested as a cutoff point to clarify unclear cases. 

It could be best to have the basal volumes to compare with volumes during rejection. But usually this is not the case and so we considered this control group as normal. Another limitation is its retrospective design. In conclusion, kidney volume determination can be a valuable tool to differentiate various causes of acute rejection. It is useful in not very clear descriptions, hence, it helps the clinician to a better decision making.
